# Contributions of memory and brain development to the bioregulation of naps and nap transitions in early childhood

**DOI:** 10.1073/pnas.2123415119

**Published:** 2022-10-24

**Authors:** Rebecca M. C. Spencer, Tracy Riggins

**Affiliations:** ^a^Department of Psychological & Brain Sciences, University of Massachusetts, Amherst, MA 01003;; ^b^Neuroscience & Behavior Program, University of Massachusetts, Amherst, MA 01003;; ^c^Institute for Applied Life Sciences, University of Massachusetts, Amherst, MA 01003;; ^d^Department of Psychology, University of Maryland, College Park, MD 20742

**Keywords:** sleep, memory, development, hippocampus, brain

## Abstract

The transition from multiple sleep bouts each day to a single overnight sleep bout (i.e., nap transition) is a universal process in human development. Naps are important during infancy and early childhood as they enhance learning through memory consolidation. However, a normal part of development is the transition out of naps. Understanding nap transitions is essential in order to maximize early learning and promote positive long-term cognitive outcomes. Here, we propose a novel hypothesis regarding the cognitive, physiological, and neural changes that accompany nap transitions. Specifically, we posit that maturation of the hippocampal-dependent memory network results in more efficient memory storage, which reduces the buildup of homeostatic sleep pressure across the cortex (as reflected by slow-wave activity), and eventually, contributes to nap transitions. This hypothesis synthesizes evidence of bioregulatory mechanisms underlying nap transitions and sheds new light on an important window of change in development. This framework can be used to evaluate multiple untested predictions from the field of sleep science and ultimately, yield science-based guidelines and policies regarding napping in childcare and early education settings.

Children spend as much as half of their early years asleep (1). Sleep promotes healthy brain and cognitive development (2). However, the mechanisms underlying these relations are poorly understood. Understanding children’s sleep needs can be used to promote optimal cognitive function and is deemed a priority in sleep research (3). Additionally, whether and for how long sleep needs must be met with naps are concerns for parents and early childhood educators.

With growing investments in universal early education in the United States, there is increasing scrutiny and awareness of how time is spent in these settings in order to maximize academic success. Should children be sleeping or learning in the early education setting? Ample data in adults and children support a role of sleep in solidifying memories, and yet, the intuition is that classroom time should be spent learning, not sleeping (4, 5). Moreover, some children within the preschool age range seem unable to nap, while others, at the same age, can be hard to keep awake (6). Because there are currently no formal recommendations regarding nap timing/length or nap transitions (e.g., from the American Academy of Pediatrics or the National Sleep Foundation), some have devalued daytime naps for children over the age of 2 y (7), even proposing the elimination of naps in publicly funded preschools (4). Related, sleep advice for parents at this age is provided by a growing number of sleep coaches; however, these individuals lack evidence-based information and guidance on such issues (8). Therefore, in order to promote optimal learning and development through sleep in infancy and early childhood, a scientific understanding of nap transitions is essential as this will inform guidelines regarding nap needs and transition points.

The need to nap is driven by the bioregulation of sleep and wakefulness (9). Sleep regulation, which can be thought of as “the drive to sleep when we do,” underlies the need for naps as well as the ability to adjust to schedule changes (e.g., daylight savings, jetlag). Disorders of sleep regulation result in insufficient sleep, which is associated with depression and anxiety, even in childhood (10). Sleep regulation is also altered in neurodevelopmental disorders (e.g., autism, Down syndrome, Attention-deficit/hyperactivity disorder or ADHD) (11, 12). Here, we describe what is known regarding the bioregulation of sleep in early development. We first discuss the relation between nap transitions and sleep regulation, including underlying neural and physiological mechanisms. Next, we consider how changes in the underlying neural substrates are related to changes in early learning and memory. Taken together, these data lead to our novel prediction that nap transitions are driven by changes in memory that are related to brain development.

## 1. Nap Transitions in Infants and Children

Newborns sleep up to 20 h each day, with sleep distributed across multiple sleep bouts (polyphasic), largely reflecting the need to feed frequently. Overnight sleep becomes distinct from daytime naps between 4 and 6 mo in most infants, but sleep remains polyphasic, with three or more naps in addition to the overnight sleep bout (13).

Sleep becomes triphasic by 9 mo of age, consisting of two daytime naps and an overnight sleep bout (14). Between the first and second years of life, the morning nap fades, and sleep becomes biphasic (1, 14). The transition to adult-like monophasic sleep most commonly occurs in the early childhood years (13–15). However, there is significant variability in the timing of the transition from triphasic and biphasic sleep (ranging from 6 to 18 mo) and even more variability in the transition out of biphasic sleep (which can be as early as 2 y and as late as 8 y of age) (13, 16).

We opt to use the terminology “nap transition” (as opposed to “nap cessation”) to capture the fact that changes in nap patterns are not instantaneous. A longitudinal study of early nap transitions (17) and meta-analyses of cross-sectional data support that transitions take place over a number of months, with naps gradually reducing in frequency and length over time (6, 13, 15). Central to our research is that these sleep transition periods are interesting windows of change in brain and memory development.

## 2. Bioregulation of Sleep and Naps

### 2.1. Bioregulation of Sleep.

The two-process model of sleep regulation is a prominent conceptual model, which posits that sleep regulation emerges from the interaction of two distinct biological processes (18, 19). The first process is the circadian control of sleep, termed Process C, which underlies the 24-h pattern in sleep behaviors. The second process is the homeostatic regulation of sleep through a mechanism termed Process S or the homeostatic sleep drive. This is the process that allows for the self-regulation of sleep, adjusting to changing conditions while preserving sleep.

#### 2.1.1. Circadian regulation of sleep (Process C).

Underlying Process C is a central circadian pacemaker that synchronizes multiple physiological processes to the same 24-h rhythm. Located in the suprachiasmatic nucleus (SCN) of the anterior hypothalamus, the circadian pacemaker is entrained to external cues or zeitgebers. Social cues, such as routine mealtimes and exercise, can serve as zeitgebers (20). However, the most common zeitgebers are light/dark cues. The SCN signals the pineal gland to synthesize the hormone melatonin on a circadian schedule. Although melatonin does not promote sleep per se, it is released from the pineal gland in darkness and reciprocally inhibits the SCN, dampening its alertness-promoting actions and ultimately, demoting wakefulness (21).

#### 2.1.2. Homeostatic regulation of sleep (Process S).

If left to the circadian system alone, we would fall asleep at the same time and wake up at the same time regardless of sleep history; overnight sleep timing would be the same following a night of poor sleep and following a luxurious afternoon nap (one not taken to compensate for sleep loss). Rather, the common experience is that it is tempting to fall asleep much earlier following a night of poor sleep and hard to fall asleep at the usual time following an afternoon nap. This reflects the second aspect of sleep regulation, Process S or the homeostatic mechanism that drives sleep. Homeostatic sleep pressure accumulates across periods of wake and dissipates with subsequent sleep (19). The mechanism underlying homeostatic control is complex, and current evidence suggests that it may be a distributed process across multiple pathways (reviewed in ref. 22).

A neurobiological marker of homeostatic sleep pressure is slow-wave activity (SWA), activity in the delta-frequency band (0.5 to 4.5 Hz) in the sleep electroencephalogram (EEG) thought to be cortically generated (23). SWA reflects the frequency of the high-amplitude slow waves characteristic of slow-wave sleep. SWA is highest early in a sleep bout and dissipates across the sleep bout. Moreover, SWA is higher following sleep deprivation, supporting the use of SWA changes as a measure of the accumulation of homeostatic sleep pressure (24). Wake-related increases in SWA may result from global increases in synaptic strength related to learning. Slow waves emerge from bistability of cortical neurons in non-rapid eye movement (non-REM) sleep, oscillating between wake-like tonic firing (up state) and neuronal silence (down state) (25). Following long periods of wake, increased global synaptic strength may contribute to higher neural synchrony or a greater number of neurons contributing to the oscillation. Collectively, there is clear support that SWA is functionally involved in maintaining sleep homeostasis, but little more is understood regarding this mechanism.

### 2.2. Bioregulation of Naps and Nap Transitions in Early Childhood.

Naps and nap transitions are influenced by multiple factors, including genes (although minimally) (17, 26), environment (27, 28), and culture (29–31). The two-process model provides a framework to understand how these factors exert their influence on sleep. For example, parents may choose to promote a consistent nap time (28), reflecting circadian contributions to nap patterns. However, many children will nap even in the absence of nap promotion, and some children are unable to nap even in sleep-promoting conditions (6), likely reflecting homeostatic processes (i.e., variability in the accumulation of homeostatic sleep pressure).

#### 2.2.1. Circadian contribution to nap patterns.

Consistency of nap times is recommended as it is thought to promote stable and predictable sleep patterns (32). As such, parents and childcare providers institute many zeitgebers (such as light availability), which may then reinforce a circadian drive to sleep at these times during the day. There is evidence that these changes in environment alter the onset of melatonin and ultimately, timing of sleep (33). Conversely, parents with negative views toward naps in this age group likely provide no zeitgebers or no nap opportunity, and as such, those children have fewer naps and naps of shorter duration (28). Cultural variation in naps may likewise reflect the use of light and rule setting, which may promote or demote daytime sleep (16, 33).

#### 2.2.2. Homeostatic contributions to nap patterns.

Whereas parent-guided zeitgebers can shift the circadian timing of sleep bouts, homeostatic control of sleep offers an explanation as to why naps can result from late bedtimes. Late bedtimes are often followed by too-early wake times due to parent or childcare schedules. The resulting shortened overnight sleep creates additional sleep pressure or the need to nap the subsequent day. However, while this provides an explanation of how environmental and cultural influences contribute to the presence of napping, they do not provide an obvious explanation of how and when nap transitions may occur. In other words, they do not explain the persistence of napping.

Nap transitions have most commonly been ascribed to Process S (34, 35). Specifically, more rapid accumulation of homeostatic sleep pressure in young compared with older children is thought to create the need to more frequently nap to release this sleep pressure. Preliminary support of this comes from a study of a small sample (*n* = 8) of 2- to 5-y-old children (36). Sleep pressure was varied by altering the amount of time that the child was awake before a nap was permitted (4, 7, or 10 h after waking). Consistent with the view that SWA serves as a proxy for sleep pressure, SWA in the nap was greater following longer intervals awake. However, this difference dissipated with age; the difference in SWA across conditions was less in younger compared with older children. This suggests that sleep pressure accumulates more rapidly in young children, who are also more likely to be habitual nappers.

**Fig. 1.**
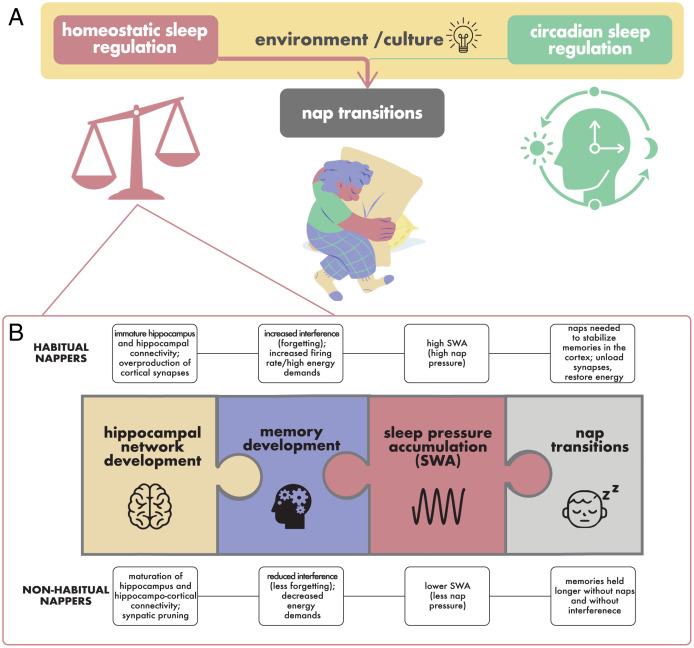
Understanding nap transitions through the two-process model of sleep regulation. (*A*) Although environmental and cultural factors (yellow; e.g., caregiver schedules and the use of light) may influence the presence of naps, they are unlikely to explain the persistence of naps and nap transitions. Rather, nap transitions are posited to largely be related to homeostatic processes (indicated by the pink arrow), with greater accumulation of sleep pressure in habitually compared with nonhabitually napping children. (*B*) We hypothesize that brain development and memory development underlie this difference in homeostatic sleep pressure. Specifically, as the hippocampal-dependent memory network develops, memories can be held for longer without interference, making more space and/or using less energy resources, which may (directly or indirectly) yield sleep pressure as measured by SWA and result in nap transitions.

In sum, there is evidence that both circadian (Process C) and homeostatic (Process S) sleep regulation processes contribute to whether a child naps (Fig. 1*A*). To date, most research regarding these processes and individual differences in nap transitions has focused on external factors, such as the extent to which parents choose to promote napping (28), a factor that varies across cultures and socioeconomic groups (27, 29). Despite these external influences, many children will nap even in the absence of nap promotion, and some children are unable to nap even in sleep-promoting conditions (6). These differences likely stem from internal processes and reflect variability in the accumulation of homeostatic sleep pressure (35). However, the biological mechanisms underlying these internal processes remain unclear. Specifically, what underlies the accumulation of homeostatic sleep pressure, and why does it vary developmentally? Additionally, how do these developmental changes contribute to changes in accumulation of homeostatic sleep pressure and ultimately, the transition out of naps? In the following section, we present a novel hypothesis that may provide answers to these questions by linking what we currently know about nap transitions with what we know about the development of the brain and its capacity to learn and remember at an early age.

## 3. A Novel Hypothesis of Nap Transitions

A unique problem exists early in human development; there is a massive amount of information that must be learned, yet the neural systems that support learning are still under construction. Schema and semantic networks that support learning in adults (37, 38) are nascent at very young ages and must be built from scratch. Moreover, the structural and functional neural circuitry that supports memory is also immature. Specifically, the hippocampus—a brain region associated with learning and memory—undergoes a period of “learning to learn” (39). Finally, to support early learning, there is overproduction of synapses across the brain, which is most prolonged in prefrontal or higher cortical areas. The increased firing rate that accompanies high synaptic density escalates energy demand (40).

Seen in this light, naps provide a solution to what is essentially a memory problem; high learning demands on an immature system create an overproduction of synapses that reaches its peak in early childhood. Sleep has been argued to help meet space and energy challenges associated with development (40, 41). Thus, we posit that early childhood is a time of competing demands of learning, which loads the brain (the hippocampus in particular), and sleeping, which may unload synapses across the cortex (42) and free up the hippocampus for ongoing learning (43).

This leads to our hypothesis that maturation of the brain, particularly the hippocampal-dependent memory network, during early childhood results in more efficient memory storage, which reduces the buildup of homeostatic sleep pressure (as reflected by SWA) and eventually, contributes to nap transitions (Fig. 1*B*). In the following sections, we provide motivation for this novel hypothesis and review initial support for this view.

### 3.1. Role of the Hippocampus in Learning and Memory in Early Development.

The ability to learn novel information and recall it later relies on a network of brain regions, including the hippocampus and neocortex. Together, these regions provide “complementary learning systems” that allow for the rapid learning of new information while both preserving existing knowledge and integrating new knowledge into these existing frameworks (41). The hippocampus is particularly important for the early stages of memory, including formation and consolidation (or stabilization) (44). In short, this structure provides short-term storage and initially works together with the neocortex to support memory of new information across long delays. Over time, connectivity among distributed cortical regions strengthens, and the role of the hippocampus gradually declines. Although learning initiates memory processes in the hippocampus, these memory traces are vulnerable to interference and forgetting. Once memories are consolidated and stabilized in the cortex, they are more robust against disruption (41).

The hippocampus is a complex structure composed of multiple subfields (cornu ammonis [CA] areas 1 to 4, dentate gyrus, and subiculum) that are distributed disproportionately along its longitudinal axis (head, body, tail) (45, 46). These regions show protracted development as a result of prolonged neurogenesis, synaptic growth, dendritic arborization, pruning, vascularization, and myelination (47–49). Data from nonhuman primates (e.g., ref. 50) and human children (e.g., ref. 51) suggest that the developmental trajectory of these subfields and their connectivity with each other are related to age-related improvements in memory, which is consistent with theoretical proposals of brain and memory development (52). Specifically, within the hippocampus, although immature cells continue to accrue within the dentate gyrus throughout the first year of postnatal life [and may be related to the onset of other cognitive abilities, such as spatial navigation (50)], elevated rates of dendritic development and synapse formation persist until at least 5 y of age (53, 54). During early childhood (∼3 to 5 y of age), neuronal connections between granule cells of the dentate gyrus and pyramidal neurons of Ammon’s horn form, which alter the functional circuits of the hippocampus (54) and regions located downstream from the dentate gyrus, particularly CA3 (50). Because circuitry in the dentate gyrus is critical for adult-like memory formation, its protracted developmental profile suggests that adult-like memory formation in humans may not be expected before 5 y, as morphological development is likely correlated with functional capability (54).

Prolonged development of memory due to the long maturational time line of neural circuitry is supported by behavioral research. Specifically, as children mature, they are better able to remember individual items as well as associations between items (55, 56). In fact, children’s ability to remember such details shows accelerated rates of change between 5 and 7 y of age (55). These findings fit well with cross-sectional research suggesting that children’s ability to bind information increases between 4 and 6 y of age (e.g., refs. 57–59). Moreover, these improvements in memory are associated with variations in hippocampal development (as indexed by volume). Across two different studies and two different memory paradigms, brain–behavior relations have been found to change around 6 y of age—following the final nap transition. Specifically, in younger (4- to 6-y) children, better memory performance is associated with larger hippocampal subfield volumes, whereas the opposite is true in older (6- to 8-y) children (i.e., better memory performance is associated with smaller volumes). This difference in brain–behavior relations may be the result of synaptic pruning within the dentate gyrus → CA3 → CA1 circuit, which is critical for binding and ultimately, yields a more efficient system (50, 54).

**Fig. 2.**
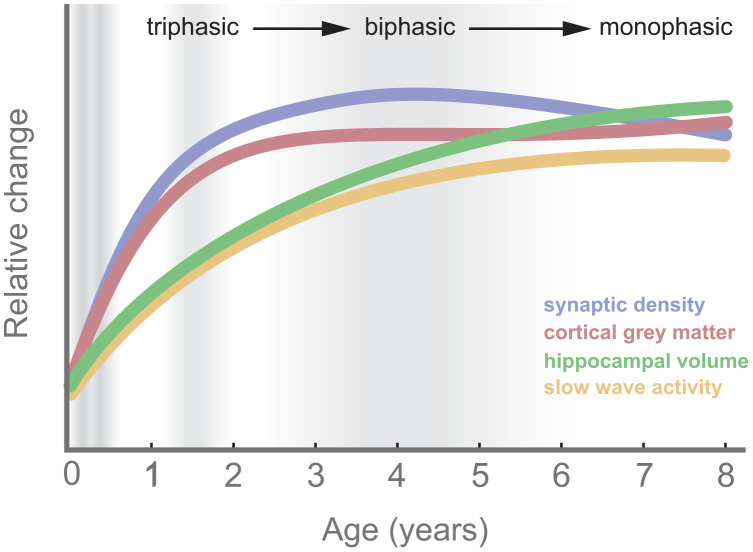
Growth trajectories across nap transitions (gray shading). Synaptic density [prefrontal cortex (113)], hippocampal volume (114, 115), cortical gray matter (116), and SWA (117) are illustrated.

In summary, ample evidence supports the role of the hippocampus (and its associated neocortical network) in learning and memory even from an early age [e.g., infants 3 to 24 mo (60)]. Critical to the present hypothesis, across early childhood, the hippocampus is learning to learn in conjunction with protracted development, a challenge that we posit is met by naps (Fig. 2).

### 3.2. SWA Supports Hippocampal-Dependent Memory.

#### 3.2.1. Evidence of SWA-related memory processing in adults.

Memories from the day benefit from subsequent sleep. Recall is better following sleep compared with an equivalent interval awake, with memories protected by the sleep interval compared with the decay that typically occurs over wake. For example, in one study, adults retained memory for more word pairs following an interval with sleep compared with an equivalent interval awake (61). Notably, memory retention was associated with time spent in slow-wave sleep, suggesting an active role of this sleep stage in memory processing as opposed to passive protection of memories during the sleep interval as a whole. Further support for the role of slow-wave sleep is provided by studies that represent auditory or odor cues associated with learned material during subsequent slow-wave sleep, which results in enhanced consolidation of cue-related material (62). Conversely, when SWA, the primary spectral power of slow-wave sleep, is reduced, performance benefits following sleep are absent (63). Such results support that consolidation of memories is enhanced by sleep and that SWA in particular supports memory consolidation.

Notably, not all memories benefit from sleep. Rather, engagement of the hippocampus during encoding seems to be a necessary condition for sleep-dependent memory consolidation (64–66). For example, memory for novel objects alone is not reliant on the hippocampus, whereas memory requiring binding of an object with a location or ordering with another object does (67). When rats performed a task requiring memory for novel objects (“what” memory), object location (“where” memory), or temporal order (“when” memory), performance was better following an interval of sleep compared with wake for the object placement and temporal order tasks. Memory for novel objects did not differ for sleep and wake conditions. This is taken as evidence that sleep specifically supports memory for tasks engaging the hippocampus (64).

Mechanistically, the synaptic homeostasis hypothesis contends that the association between SWA and memory consolidation over the sleep interval reflects synaptic downscaling over sleep, with superfluous synapses (incidental memories) being downscaled, while intentionally learned memories are benefited by reduced signal to noise (38). According to the active systems consolidation view, synchronous slow waves aligned with hippocampal activity (i.e., hippocampal sharp-wave ripples) support a hippocampal–neocortical shift of the memory representation. Numerous studies in animals have demonstrated that patterns of hippocampal activation associated with a learning episode are seen again in subsequent sleep and not in the prior sleep bouts (68). Such “replay events” undergo developmental changes early in life (69) and have been linked with the emergence of episodic-like memory in rodents (70). The timing of these developmental changes coincides with the transition to monophasic sleep in humans [i.e., roughly during the early childhood years (13–15)]. Replay activity is associated with hippocampal ripples, which co-occur with sleep spindles and slow oscillations (71). Although hippocampal ripples cannot be measured noninvasively in humans, spindle–slow oscillation coupling predicts performance changes over sleep in adults (72, 73). Growing support for both the synaptic homeostasis hypothesis and the active systems consolidation view has brought many to agree that these memory-supporting mechanisms in sleep are not mutually exclusive (74, 75). Although both of these theories have influenced our thinking, our proposal intentionally does not ascribe to either one specifically, nor is our work intended to provide direct evidence supporting one or the other. Rather, we focus on the importance of sleep for memory—as it both stabilizes existing memories in cortical regions and optimizes synaptic organization (76), which ultimately frees up the hippocampus for ongoing learning.

#### 3.2.2. Evidence of SWA-related memory processing in children.

Sleep benefits memory consolidation from a very young age. Benefits of sleep relative to wake have been observed in the performance of children ranging from 6 mo through adolescence, particularly for declarative learning tasks (reviewed in ref. 77). Under normal conditions, motor procedural learning does not benefit from sleep in childhood. However, when children receive additional training, performance improves following sleep relative to wake (78), which may suggest that, like adults, hippocampal engagement with encoding may be essential to sleep-dependent memory consolidation. Moreover, a study in toddlers supports that hippocampal-based memories are reactivated during a nap even in children as young as 2 y (79), providing evidence that the hippocampus is functional during sleep and directly related to memory at an early age.

Interestingly, SWA in children’s naps predicts the overnap protection of memory for emotional faces (80) and also, predicts reductions in the emotional attention bias following a nap in young children (81). We posit that consolidation of emotional memories from the morning decreases emotional load, and as a result, children are less reactive to emotional stimuli thereafter. This provides a potential explanation of the oft observed phenomena that habitually napping children are emotionally dysregulated at the end of the day if they do not nap (the “witching hour”).

Sleep-related declarative memory benefits in children have also been associated with sleep spindles and accompanying slow oscillations in children 3 to 5 y (82, 83). Moreover, a longitudinal study of older children (9 y) who were tested subsequently at 16 y of age showed that developmental increases in coupling strength between spindles and slow oscillations provided a strong predictor of developmental changes in sleep-related benefits on a word-pair learning task (84). This provides additional support for the cortical stabilization of memories, initially encoded in the hippocampus, in the cortex during sleep and the theory that this process is present in children and strengthens with development.

Notably, whether such benefits reflect hippocampal–neocortical transition of memory representations as proposed by the active systems consolidation theory, cortical stabilization via synaptic downscaling as proposed by the synaptic homeostasis hypothesis, or both is unknown. It has been hypothesized that active systems consolidation would be unlikely before 18 to 24 mo due to underdevelopment of the hippocampus (85). Although few studies in this young age group have included polysomnography (77), there is some evidence that sleep-dependent memory processing is associated with sleep spindles, even at this young age. For instance, 9- and 16-mo-old infants who learned word–object pairs prior to a nap or wake interval generalized to category exemplars only after sleep. Notable here is that nap-related generalization was associated with the EEG sigma power, the frequency range of sleep spindles (10 to 15 Hz) (86, 87). This lends support that key brain areas for active systems consolidation may be sufficiently developed in infancy (60). An alternative is that synaptic downscaling oversleep accounts for memory benefits at a very young age, while active systems consolidation accounts for sleep-related benefits later on (85). Importantly, the present hypothesis can accommodate either mechanism.

### 3.3. Evidence Supporting the Relation between Brain Development and Nap Transitions.

Support for the present hypothesis can be drawn from the coincident development of brain, memory, and sleep described above. Additionally, a growing number of behavioral and neural developmental studies provide more direct support for the relation between brain development and nap transitions, although to date, this work has been focused on the biphasic to monophasic sleep transition.

#### 3.3.1. Behavioral studies.

At a behavioral level, support for relations between brain development and nap transitions can be drawn from studies comparing cognitive performance in habitually and nonhabitually napping children who are the same age. In one such study, children who performed better on a cognitive battery were found to take fewer naps than those with lower performance (88). In particular, fewer naps corresponded to enhanced memory span for auditory number sequences and larger vocabulary. One interpretation of this result is that those who nap have insufficient overnight sleep (supported by a negative correlation between nighttime sleep and nap length), and this may explain both lower cognitive performance and nap frequency. However, an alternative is that nap habituality is related to brain maturation; children who have more mature brains need to nap less often and also, perform better on cognitive assessments.

Supporting the latter interpretation, our recent study provided experimental evidence of differences in memory performance around the nap transition. In this study, we taught habitually napping and nonhabitually napping children a visuospatial task prior to an afternoon nap and again prior to an equivalent interval awake (within subjects) (82). Memory for item locations was probed again after the nap or wake interval and once more the following morning. We found that memories were protected by the nap; accuracy following the nap did not differ from immediate recall. However, when children stayed awake during the nap opportunity, recall accuracy was reduced by ∼12% compared with immediate recall. The benefit of the nap remained when performance was assessed again the following morning. We then considered whether the nap benefit varied for children who napped habitually (greater than or equal to five naps per week) compared with those who no longer napped (zero to time per week but were nap promoted for the experiment). Memory consolidation over the nap did not differ for habitually and nonhabitually napping children; naps protected memories regardless of nap habituality. Rather, what differed was how much memories decayed over the waking interval; memory decay over an afternoon awake was greater for children who napped habitually and minimal for those who no longer napped, even when controlling for age. We interpret this as evidence that in habitual nappers, sleep needs to occur more frequently in order to prevent the catastrophic interference between memories that occurs when kept awake. Nonhabitual nappers, on the other hand, may have more developed memory storage and thus, be able to hold memories for longer without interference. Subsequently, other studies have found similar differences between habitually and nonhabitually napping children with a word-learning task (89) and an emotional face-learning task (80). Collectively, these studies provide evidence that naps are similarly beneficial regardless of nap habituality but that memory of habitually napping children is much more damaged by a missed nap compared with nonhabitually napping children.

#### 3.3.2. Neural studies.

At a neural level, SWA, the marker of the accumulation of sleep pressure that contributes to naps (36, 90), has also been related to brain development (as reviewed in ref. 91). Slow-wave amplitude increases during childhood and is highest shortly before puberty. This parallels findings of developmental changes in synaptic density (Fig. 2) (92). SWA is highly predictive of decreases in gray matter volume, a relation that is strongest in areas undergoing maturation at this age (93). In addition, the distribution (or topography) of SWA also tracks with the development of underlying cortical areas (93, 94). Specifically, SWA, which peaks maximally over occipital regions in younger children, shifts to a peak over frontal regions by adolescence, a trajectory that mirrors that of cortical maturation [cortical thickness (93, 94)] and is predictive of brain myelin (95). Taken together, these studies support a link between brain development and global SWA in early childhood.

Finally and more directly, we recently reported a difference in hippocampal volume for habitually and nonhabitually napping children. We compared hippocampal subfield volumes in 4- to 6-y-old children who napped habitually with those who did not nap habitually. Habitually napping children had larger CA1 hippocampal subfield volumes in the hippocampal body compared with nonnapping children (96). This study provides the first direct evidence for a difference in the hippocampus between habitually napping and nonhabitually napping children that cannot be accounted for by age. Critically, prior reports on this same sample of children have linked volume of the CA1 to children’s memory performance, and this region also shows developmental change across the early childhood period (51, 97). Most germane to our hypothesis is the finding that across all children in the study (4 to 8 y, regardless of nap status), smaller CA1 was associated with better memory performance (53).

### 3.4. Summary, Caveats, and Future Research Directions.

Collectively, we provide support for a relation between nap transitions and underlying memory and brain development. Together with studies relating nap habituality to SWA and memory to SWA, we provide a parsimonious hypothesis suggesting that maturation of the hippocampal-dependent memory network during early childhood results in more efficient memory storage, which reduces the buildup of homeostatic sleep pressure and in turn, contributes to nap transitions (Fig. 1*B*).

However, there are some caveats given the limited body and nature of the current literature. First, much of the data supporting our hypothesis lack causality. While we posit that reduced need for memory consolidation is related to changing accumulation of sleep pressure (and related SWA), whether these are related directly (e.g., SWA changes could be related to simultaneous development in cortical regions in the hippocampal–memory network where SWA may be generated) or indirectly (e.g., less adenosine accumulation in the developing hippocampus benefiting memory and less adenosine accumulation in the cortex reducing SWA) is important to consider. We also interpret associations between the development of the hippocampal-dependent memory network and sleep, particularly nap habituality, to indicate that development of the brain drives nap habituality. An alternative developmental time line is that changes in sleep are a necessary precursor to brain development. Indeed, changes in SWA have been shown to precede improvements in some motor skills, which in turn, preceded decreases in gray matter in a cross-sectional sample of those 2 to 26 y of age (98). It will also be important to consider the development of other brain regions associated with the bioregulation of sleep. For example, hypothalamic development may follow a similar time line (99). Although this is unlikely to account for the differences in behavior of habitual and nonhabitual nappers described above, it will be important for future work to consider interactions in the development of multiple brain areas, which may support the bioregulation of sleep in early development and their causal role in nap habituality.

Second, while we posit that brain and memory development may underlie the transition from triphasic to biphasic sleep, current data in support of the hypothesis are based on the biphasic to monophasic sleep transition. Moreover, the present hypothesis is based on mostly cross-sectional data supporting individual relationships between sleep–brain, sleep–memory, or brain–memory. It is essential to acquire longitudinal data that capture sleep physiology, structural and functional brain development, and memory changes across the nap transitions, starting at 12 mo to capture both critical transitions, within a single sample that is large enough to overcome individual differences and tease apart general maturation from nap-related change. Such longitudinal data can be probed using latent change score modeling to assess the relations between these factors. Latent change score models evaluate ways in which variables are recursively associated over time in order to isolate temporal components of change within a person or group in order to specify lead vs. lag (100). For example, latent change score modeling has been used successfully in studies of memory decline to better understand the dynamics among processes, including changes at the neural level (e.g., ref. 101).

Third, there is considerable variability in the experimental methods used to examine the impact of sleep on memory early in life. This variation applies to the stimuli used (words vs. pictures), age ranges explored, and durations over which information is retained, as well as other factors. These differences likely contribute to the lack of consensus in results. For example, one study that used a verb-learning task, which required generalization to a novel exemplar, failed to find a difference in performance between habitual and nonhabitual nappers following intervals of wake and sleep (which benefited both groups) (102). However, it is possible that measurement limitations (as change in performance over the interval cannot be assessed) or other task differences may account for these discrepancies. Moreover, there are clear task nuances that can vary sleep’s function in memory consolidation even in the adult literature (103, 104). Better understanding of these tasks and related neural underpinnings is essential for a mechanistic understanding of nap function and nap transitions. Thus, it is important for future work to also consider memory consolidation with various types of tasks as it relates to brain development.

Finally, several factors contribute to the presence of naps. Cultural (including parenting practices) and environmental factors likely play a role (105–107). Although many of these are related to circadian and homeostatic sleep regulation (section 2), many other factors could be considered [e.g., diet, illness (108)] that are likely to contribute to the presence of naps but not the persistence of naps and nap transitions. Importantly, while we posit that brain maturation is a strong factor underlying nap transitions, these factors are not mutually exclusive [e.g., environmental factors can also contribute to brain development (109)]. Moreover, whether other species demonstrate similar nap transitions and factors that may affect these transitions have received little attention and would contribute greatly to a broader understanding of sleep regulation. Identifying factors that moderate or interact with brain development and sleep is an important goal for future research.

## 4. Conclusions and Implications

Here, we present a first review of the bioregulatory processes that contribute to the napping patterns of young children. Every young child naps and transitions out of naps at some point in early childhood—but the age at which this transition takes places varies dramatically (14, 15, 110).

Better understanding nap transitions would allow educators and caregivers to support these transitions, thereby strengthening children’s health and cognition. Scientific evidence showing that nap transitions are a product of brain development that is quite variable between individual children would help parents and providers appreciate that nap transitions cannot be determined by age and that the opportunity to nap should be protected for those that need it. This would also have substantial policy implications for early education, suggesting that nap opportunities should not only be protected for some children but actively supported and of sufficient length. Moreover, understanding typical sleep development is essential to identifying disorders of sleep as well as the extent of the impact associated with insufficient sleep. Thirty percent of children aged 6 to 11 y have insufficient sleep (111). Unfortunately, the extent of this deficiency in younger children is unclear due to a dearth of data on sleep in early childhood (1). This is egregiously problematic as younger children are at greater risk for problems stemming from insufficient sleep due to their immature brain development. Moreover, sleep is particularly reduced in low-income and atypically developing children (112) of this age and thus, contributes to known health disparities that are widespread and have far-reaching impacts. Given the prevalence of naps, the importance of naps to early cognition, and the policy ramifications, a scientific understanding is essential, as these periods may provide unique windows of opportunity to promote healthy development and optimize cognitive abilities.

## Data Availability

There are no data underlying this work.
